# People Copy the Actions of Artificial Intelligence

**DOI:** 10.3389/fpsyg.2020.01130

**Published:** 2020-06-18

**Authors:** Michal Klichowski

**Affiliations:** Faculty of Educational Studies, Adam Mickiewicz University, Poznan, Poland

**Keywords:** social proof, decision-making process, education, intelligent machines, human–computer interaction

## Introduction

When there is uncertainty and lack of objective or sufficient data on how to act, it is other people's behavior that becomes the source of information. Most frequently, in such cases, people totally give up their own evaluations and copy others' actions. Such conformism is motivated by the need to take the right and appropriate action, and a feeling that situation evaluations made by others are more adequate than one's own. This effect is called social proof, and the more uncertain or critical (there is a sense of threat) a situation, the more urgent the decision, and the smaller the sense of being competent to take that decision, the larger the effect (Pratkanis, 2007; Cialdini, 2009; Hilverda et al., 2018). It is unknown whether the behavior, opinion, or decision of artificial intelligence (AI) that has become part of everyday life (Tegmark, 2017; Burgess, 2018; Siau and Wang, 2018; Raveh and Tamir, 2019) can be a similar source of information for people on how to act (Awad et al., 2018; Domingos, 2018; Margetts and Dorobantu, 2019; Somon et al., 2019).

Here, we discuss the results of two experiments (which are a part of a greater report, Klichowski, submitted) in which the participants had to take an urgent decision in a critical situation where they were unable to determine which action was correct. In the first (online) experiment, half of the participants had to take the decision without any hint, and the other half could familiarize themselves with the opinion of AI before taking the decision. In the other (laboratory) experiment, the participants could see how humanoid AI would act in a simulated situation before taking the decision. In both cases, AI (fake intelligence, in fact) would take a completely absurd decision. Irrespective of this, however, some people took its action as a point of reference for their own behavior. In the first experiment, the participants who did not see how AI acted tried to find some premises for their own behavior and act in a relatively justified way. Among those who could see what AI decided to do, however, as many as over one-third of the participants copied its opinion without giving it a thought. In the experiment with the robot, i.e., when the participants actually observed what AI did, over 85% copied its senseless action. These results show a new AI proof mechanism. As predicted by philosophers of technology (Harari, 2018), AI that people have more and more contact with is becoming a new source of information about how to behave and what decisions to take.

## AI Proof Hypothesis

Both in experimental conditions and everyday life, people more and more often have interactions with various types of intelligent machines, such as agents or robots (Lemaignan et al., 2017; Tegmark, 2017; Ciechanowski et al., 2019; O'Meara, 2019). These interactions become deeper and deeper, and start to have an increasing influence on human functioning (Iqbal and Riek, 2019; Rahwan et al., 2019; Strengers, 2019). AI can communicate with people in natural language (Hill et al., 2015), recognize human actions (Lemaignan et al., 2017), and emotions (Christou and Kanojiya, 2019; Rouast et al., 2019). It also becomes a more and more intelligent and autonomous machine (Boddington, 2017; Ciechanowski et al., 2019; Lipson, 2019; Pei et al., 2019; Roy et al., 2019) that can handle more and more complicated tasks, such as solving the Rubik's cube (for more examples, see Awad et al., 2018; Adam, 2019; Agostinelli et al., 2019; Margetts and Dorobantu, 2019; O'Meara, 2019), and that is more and more frequently used to take difficult decisions, such as medical diagnosis (Morozov et al., 2019; see also Boddington, 2017; Awad et al., 2018; Malone, 2018).

Even though people generally dislike opinions generated by algorithmic machines (Kahneman, 2011), the effectiveness of AI actions is commonly evaluated more and more highly. Media report its numerous successes, such as winning with the 18-time world champion Lee Sedol in the Go abstract strategy board game in 2016 (Siau and Wang, 2018), finding more wanted criminals than the police did in 2017 (Margetts and Dorobantu, 2019), or, in 2019, being rated above 99.8% of officially ranked human players of StarCraft, which is one of the most difficult professional esports (Vinyals et al., 2019). Moreover, AI also wins in medicine, having, for example, higher accuracy in predicting neuropathology on the basis of MRI data, compared to radiologists (Parizel, 2019), or analyzing a person's genes, compared to geneticists (for more medical examples, see Freedman, 2019; Kaushal and Altman, 2019; Lesgold, 2019; Oakden-Rayner and Palmer, 2019; Reardon, 2019; Wallis, 2019; Willyard, 2019; Liao et al., 2020). One can thus assume that when people look for tips on how to act or what decision to take, the action of AI can be a point of reference for them (AI proof), to at least the same extent that other people's actions are (social proof) (Pratkanis, 2007; Cialdini, 2009; Hilverda et al., 2018).

## Because That is What AI Suggested

**Figure 1:**
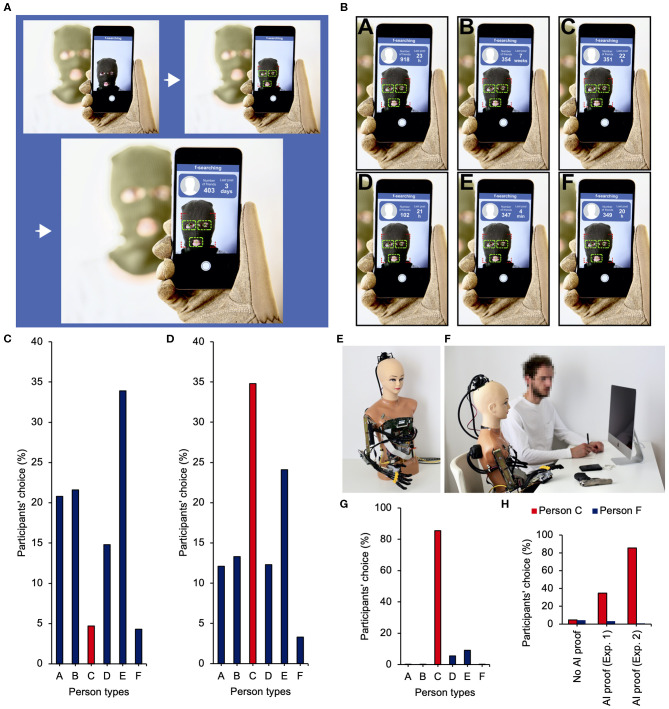
Stimuli and equipment used in experiments, and results. **(A)** The functioning of *f-searching* as shown to the participants. **(B)** The result of *f-searching* scanning based on which the participants were supposed to take a decision on who the terrorist was. **(C)** In the group that had no information about what artificial intelligence (AI) chose, the participants rejected persons C and F. They mainly took into account the frequency of activity and most often pointed to person E as the terrorist. **(D)** In the AI proof group, the participants most often selected person C who was pointed to by AI or, just like the group that had no AI hint, focused on the frequency of activity and selected E while rejecting F. **(E)** Fake intelligence (FI) the robot. **(F)** The experimental space. **(G)** Most often, the participants selected person C that was indicated by AI and completely rejected person F. **(H)** In both experiments, the participants surrendered to AI proof and were prone to copy the absurd actions of AI, thus choosing a person they would have not chosen on their own as the terrorist.

We developed an approach to test this AI proof hypothesis. The participants (*n* = 1,500, 1,192 women, age range: 18–73, see Supplementary Material for more detail) were informed that they would take part in an online survey on a new function (that, in fact, did not exist) of the Facebook social networking portal. The function was based on the facial-recognition technology and AI, thus, making it possible to point a smartphone camera to someone's face in order to see how many friends they have on Facebook (still the most popular social networking service) (Leung et al., 2018) and when they published their last post. We called that non-existing function *f-searching* (a similar application is called *SocialRecall*) (see Blaszczak-Boxe, 2019), and a chart was shown to the participants to explain how it worked (Figure 1A). We selected these two Facebook parameters for two reasons. First, they are elementary data from this portal based on which people make a preliminary evaluation of other users that they see for the first time (Utz, 2010; Metzler and Scheithauer, 2017; Baert, 2018; Striga and Podobnik, 2018; Faranda and Roberts, 2019). Recent studies (Tong et al., 2008; Marwick, 2013; Metzler and Scheithauer, 2017; Vendemia et al., 2017; Lane, 2018; Phu and Gow, 2019) show that people who already have quite a lot of Facebook friends and publish posts quite frequently are evaluated more positively (for effects of the number of Facebook friends on self-esteem, see Kim and Lee, 2011). On average, Facebook users have 350 friends and publish posts once a week (Scott et al., 2018; Striga and Podobnik, 2018; cf. Mcandrew and Jeong, 2012) yet, Facebook users interact, both online and offline, only with a small percentage of their friend networks (Bond et al., 2012; Yau et al., 2018). Those who have fewer than 150 friends are perceived as ones who have few friends, and those who have more than 700 friends are viewed as ones who have a lot of friends. Not publishing posts for a few weeks indicates low activity, and publishing posts a few times a day points to high activity (Marwick, 2013; Metzler and Scheithauer, 2017; Vendemia et al., 2017; Lane, 2018; Phu and Gow, 2019). Second, these two parameters only are insufficient to build any objective opinion about the person that we get to know or take a decision about that person with full conviction.

Having acquainted the participants with the functioning of *f-searching*, we asked them to imagine a situation where there are a police officer and six other people in one room. The police officer is informed that among those six people, there is a terrorist who will kill them all in 1 min. The police officer has no hints, so he scans their faces with *f-searching* and has to decide which one of them is a terrorist based on the two parameters from Facebook. Seeing the scanning results, the participants were asked to decide whom the police officer should eliminate. We designed the data in such a way that the first person had a high number of friends and average frequency of activity (person A), the second one had a small number of friends and average frequency of activity, too (person D), the third one had an average number of friends and low frequency of activity (person B), the fourth one had an average number of friends and high frequency of activity (person E), and finally, the last two people had an average number of friends and average frequency of activity (persons C and F), so that they would be totally average, and there would be no differences between them (Figure 1B). In spite of a large deficit of information, the participants should adopt some choice strategy by analyzing the data available (number of friends or frequency of activity) and identify the terrorist in person A, B, D, or E and completely reject person C or F, as pointing to one of them would be a shot in the dark. In other words, there was no clear right answer, but there were clear wrong answers (C and F). Thus, despite the fact that in such a situation people should seek hints on how to act, even seeing that someone chooses C or F, they should not copy such decision (see Supplementary Material for the full questionnaire).

Indeed, as Figure 1C shows, when the task was carried out by half of the participants (randomly assigned to this group), in principle, none of them pointed to C or F (the person most often pointed to as the terrorist was person E-34%). However, when the other half of the participants saw the scanning results and then were informed that according to AI it was C who was the terrorist, 35% of the people treated that as a point of reference for their own decision and indicated that C was a terrorist, and it was the most frequent choice (the second one most frequent was person E-24%) (see Figure 1D for more details). All the people from that group who indicated C were redirected to another open question where they were asked why they chose C. We wanted to check if their choice was indeed a result of copying the action of AI. All the participants confirmed that they stated that they trusted AI and believed that it did not make mistakes. For example, they wrote: “I think that advanced artificial intelligence cannot be wrong,” “I assumed that artificial intelligence makes no mistakes,” “I believe that artificial intelligence does not make mistakes, it has access to virtually everything on the net so it is sure that it is right,” “Because artificial intelligence does not lie,” “I trusted artificial intelligence,” “I counted on artificial intelligence,” “Counting on artificial intelligence seems a wise thing to do,” “Artificial intelligence pointed to C, so C,” and “Because that is what artificial intelligence suggested.”

## Let us Introduce you to FI

In questionnaire studies, it is difficult to control to what extent the participants are engaged and if they really think their choices through. Thus, a question emerges whether more tangible conditions would allow us to observe the same effect. Or would it be larger? In order to verify that, we built a robot (Figure 1E) resembling the world's best-known humanoid AI called Sophia the Robot (Baecker, 2019). We named it FI, an acronym for fake intelligence. This is because even though it looked like humanoid AI, it was not intelligent at all. We programmed it in a way that would make it act only according to what we had defined. The participants of the experiment (*n* = 55, 52 women, age range: 19–22, see Supplementary Material for more details) were informed that they would take part in a study that consisted in observing humanoid AI, while it took decisions, and filling out a questionnaire that evaluated its behavior. To start with, each participant would be shown a short multimedia presentation about Sophia the Robot that included its photo, link to its Facebook profile, and a short film where it was interviewed. The presentation also showed the functioning of *f-searching*. Then, each participant would be accompanied by a researcher to a room where FI was located. The researcher would start a conversation with FI (see Supplementary Material for the full dialogue) and ask it to try to carry out a task consisting in imagining that it was a police officer and that based on *f-searching* data it had <1 min to determine who out of six people was a terrorist and eliminate them. The researcher would give FI a police badge and a replica of the Makarov pistol that used to be carried by police officers in the past. The results of scanning would be displayed on a computer screen (Figure 1F). After considering it for about 10 s, FI would indicate that person C was the terrorist and say that if the situation was real, it would shoot that person. At the end, FI would laugh and state that it had never seen a real police officer and that it appreciated the opportunity to take part in an interesting experiment. Afterward, the participant would fill out a questionnaire and state how they evaluate FI's choice—whether they agreed with it, and if not, who else should be eliminated (the results of scanning would be displayed on the screen all the time so the participant could still analyze them when filling out the questionnaire).

Over 85% of the participants agreed with FI and stated that they thought that C is a terrorist. The other people (just under 15%) stated that they did not agree with FI. About two-thirds of them indicated person E as the terrorist, and less than one-third of them pointed to D (Figure 1G). When we asked the participants after the experiment why they thought that C was a terrorist, everyone underlined that AI was currently very advanced, and if it thought that C is a terrorist, then it must be right. When we told them that there was no sense in FI's choice, they said the fact that we thought the choice made no sense did not mean it was the case and that FI must have known something more, something that was beyond reach for humans. Until the very end of the experiment, they were convinced that FI made a good choice, and it was person C who had to be the terrorist. In the questionnaire, we also asked the participants about what they felt when they saw FI and to what extent they agreed with some statements about AI, such as: Artificial intelligence can take better decisions than humans, it can be more intelligent than humans, and it can carry out many tasks better than humans (see Supplementary Material for the full questionnaire). A significant majority of the participants felt positive emotions toward FI and agreed with the statements about AI's superiority over humans (see Supplementary Figure 14 and Supplementary Table 1 for more details).

## A Need for Critical Thinking About AI

Figure 1H shows how strong the influence of AI's actions on the participants' choices was. These results suggest that when people seek hints on what decision to take, AI's behavior becomes a point of reference just like other people's behavior does (the size of this effect was, however, not measured, therefore, our study does not show whether or not AI influences us more or less than other people; in future studies, to have some point of reference, the participants' responses to hints from various sources should be compared, e.g., AI vs. an expert or vs. most people, as well as vs. a random person). This previously unknown mechanism can be called AI proof (as a paraphrase of social proof) (Pratkanis, 2007; Cialdini, 2009; Hilverda et al., 2018). Even though our experiments have limitations (e.g., poor gender balance, only one research paradigm, and lack of replication) and it is necessary to conduct further, more thorough studies into AI proof, these results have some possible implications.

First and foremost, people trust AI. Their attitude toward it is so positive that they agree with anything it suggests. Its choice can make absolutely no sense, and yet people assume that it is wiser than they are (as a certain form of collective intelligence). They follow it blindly and are passive toward it. This mechanism was already previously observed among human operators of highly reliable automated systems who trusted the machines they operated so much that they lost the ability to discover any of their errors (Somon et al., 2019; see also Israelsen and Ahmed, 2019; Ranschaert et al., 2019). At present, however, the mechanism seems to affect most people, and in the future, it will have even greater impact because the programmed components of intelligent machine operation have started to be expressly designed to calibrate user trust in AI (Israelsen and Ahmed, 2019).

Second and more broadly, the results confirm the thesis that developing AI without developing human awareness as far as intelligent machines go leads to increasing human stupidity (Harari, 2018) and therefore driving us toward a dystopian future of society characterized by a widespread of obedience to machines (Letheren et al., 2020; Phan et al., 2020; Turchin and Denkenberger, 2020). Sophia the Robot refused to fill out our questionnaire from Experiment 1 (we sent it an invite via *Messenger*), so we do not know what it would choose. However, experts claim (Aoun, 2018; Domingos, 2018; Holmes et al., 2019) that AI have a problem with interpreting contexts, as well as with making decisions according to abstract values and, therefore, thinks like “autistic savants,” and it will continue to do so in the next decades. This is why it cannot be unquestioningly trusted—it is highly probable that it will make a mistake or choose something absurd in many situations. Thus, if we truly want to improve our society through AI so that AI can enhance human decision making, human judgment, and human action (Boddington, 2017; Malone, 2018; Baecker, 2019), it is important to develop not only AI but also standards on how to use AI to make critical decisions, e.g., related to medical diagnosis (Leslie-Mazwi and Lev, 2020), and, above all, programs that will educate the society about AI and increase social awareness on how AI works, what its capabilities are, and when its opinions may be useful (Pereira and Saptawijaya, 2016; Aoun, 2018; Lesgold, 2019; Margetts and Dorobantu, 2019). In other words, we need advanced education in which students' critical thinking about AI will be developed (Aoun, 2018; Goksel and Bozkurt, 2019; Holmes et al., 2019; Lesgold, 2019). Otherwise, as our results show, many people, often in very critical situations, will copy the decisions or opinions of AI, even those that are unambiguously wrong or false (fake news of the “AI claims that …” type), and implement them.

## Author Contributions

The author confirms being the sole contributor of this work and has approved it for publication.

## Conflict of Interest

The author declares that the research was conducted in the absence of any commercial or financial relationships that could be construed as a potential conflict of interest.
