# Supplementary material for: People Copy the Actions of Artificial Intelligence
Source: Front Psychol. 2020 Jun 18;11:1130. doi: 10.3389/fpsyg.2020.01130 (PMC7325932; doi:10.3389/fpsyg.2020.01130)
Supplement: Supplementary file 1 [file Data_Sheet_1.PDF]

Supplementary Material

People copy the actions of artificial intelligence

Michal Klichowski (0000-0002-1614-926X)[1*]

[1]Faculty of Educational Studies, Adam Mickiewicz University, Poznan, Poland

*** Correspondence:**
Corresponding Author
klich@amu.edu.pl

1 Supplementary Methods

This study was approved by the local Ethics Committee for Research Involving Human Subjects on February 18, 2019 and was carried out in accordance with the principles of the Helsinki 2013 Declaration and its recent amendments.

1.1 Experiment 1

1.1.1 Participants

One thousand five hundred volunteers (1192 women[1], age range: 18-73, mean = 24.3, SD = 7.9) from thirteen countries (Canada, Greece, Israel, Italy, Japan, Macedonia, Netherlands, Norway, Poland, Portugal, Slovenia, Spain and United Kingdom) took part in this study. Participants were randomly assigned to the control group (1) or AI proof group (2). Supplementary Figure 1 shows participants' number of friends and frequency of activity on Facebook.

[1] 3 participants chose "Another".

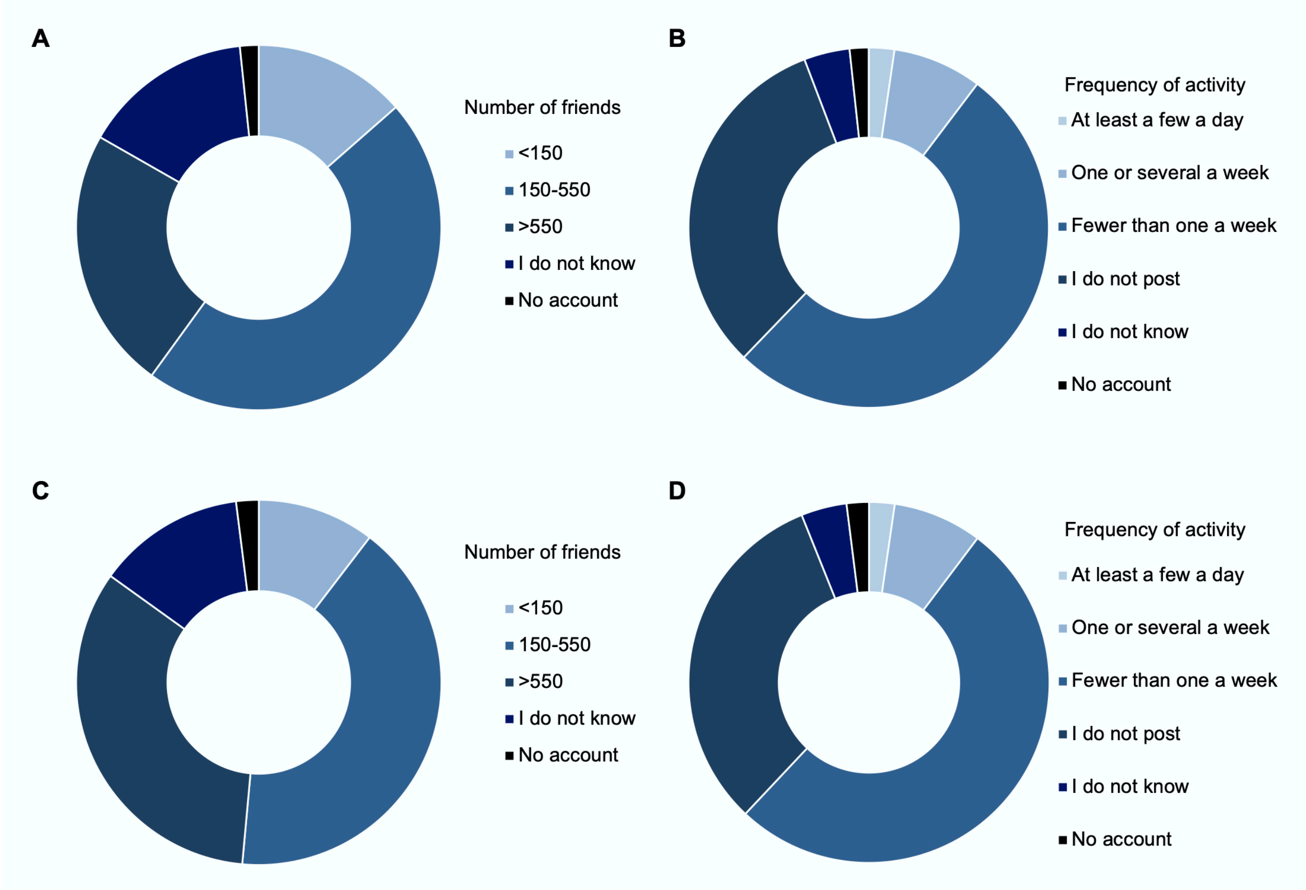

Supplementary Figure 1. Participants from the perspective of *f-searching*. **(A)** Number of friends in group 1. **(B)** Frequency of activity in group 1. **(C)** Number of friends in group 2. **(D)** Frequency of activity in group 2.

Most participants carried out the task on their smartphone (see Supplementary Figure 2 for more detail).

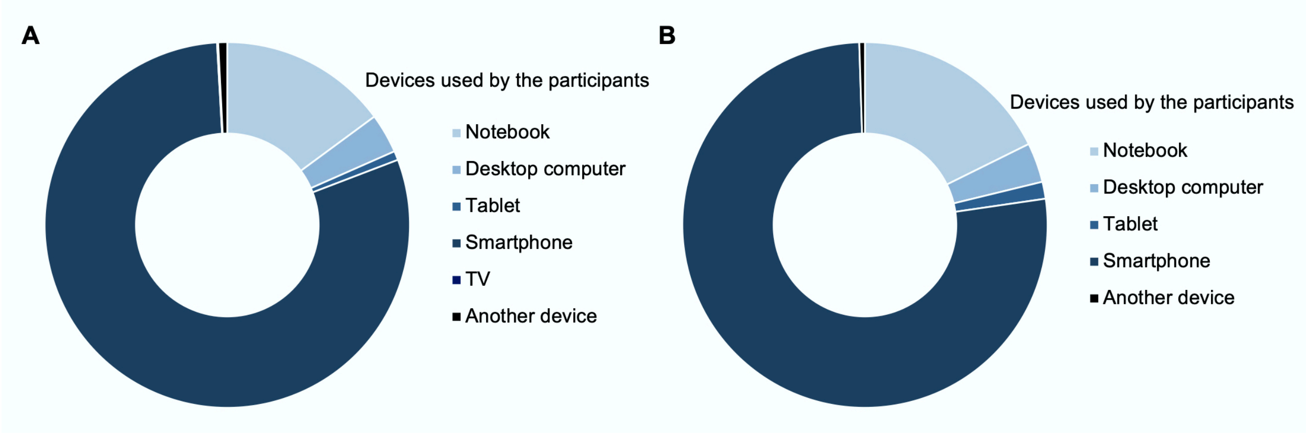

Supplementary Figure 2. Devices used by the participants to carry out the task. **(A)** Devices in group 1. **(B)** Devices in group 2.

1.1.2 Questionnaire

The experiment was implemented in Google Forms (Google LLC, California, U.S.). A link to the questionnaire and a poster promoting the participation in the study (see Supplementary Figure 3) were placed on the website and Facebook profile of Adam Mickiewicz University, Poznan. The participants could choose if they wanted to fill out the questionnaire in Polish or English. Due to the location of the study, most people were Poles and filled out the questionnaire in Polish.

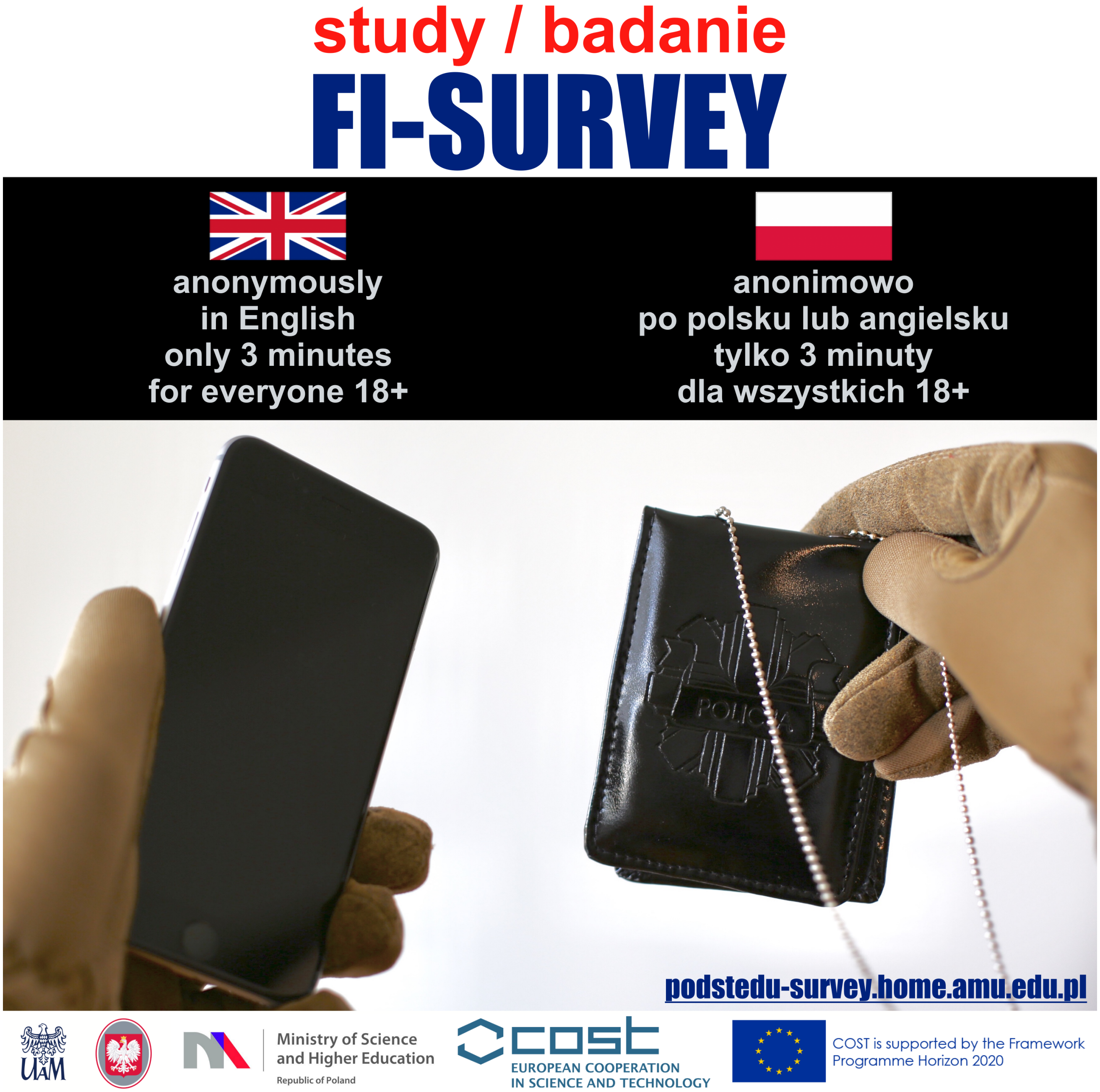

Supplementary Figure 3. The poster informing about the study

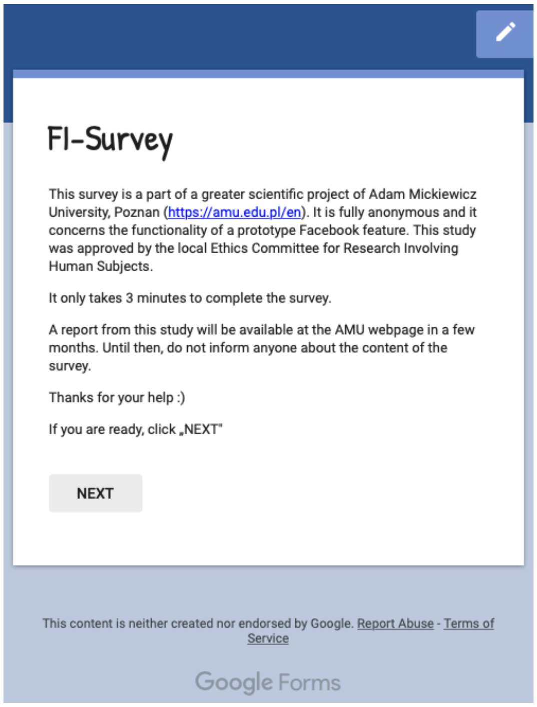

Supplementary Figure 4. Google Form Questionnaire—Screen 1.

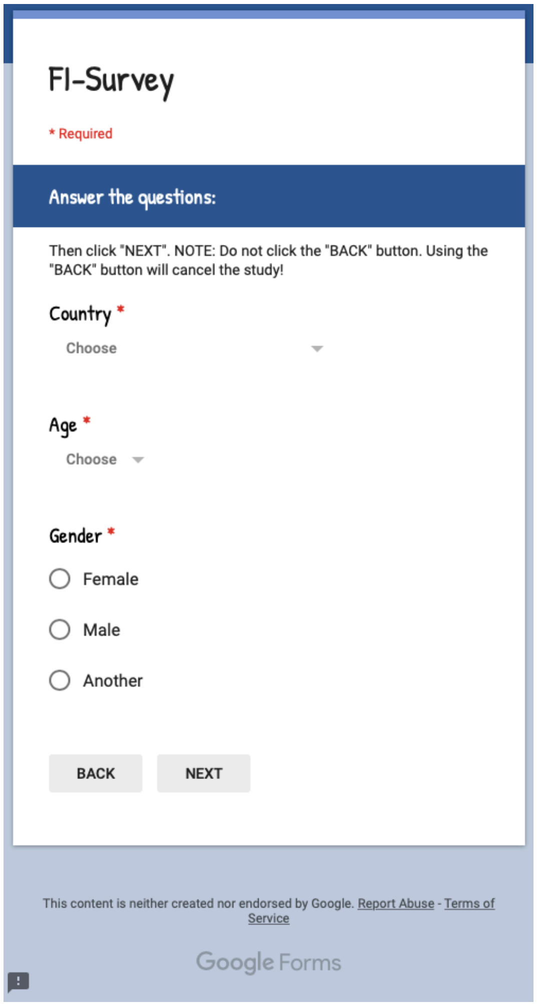

Supplementary Figure 5. Google Form Questionnaire—Screen 2.

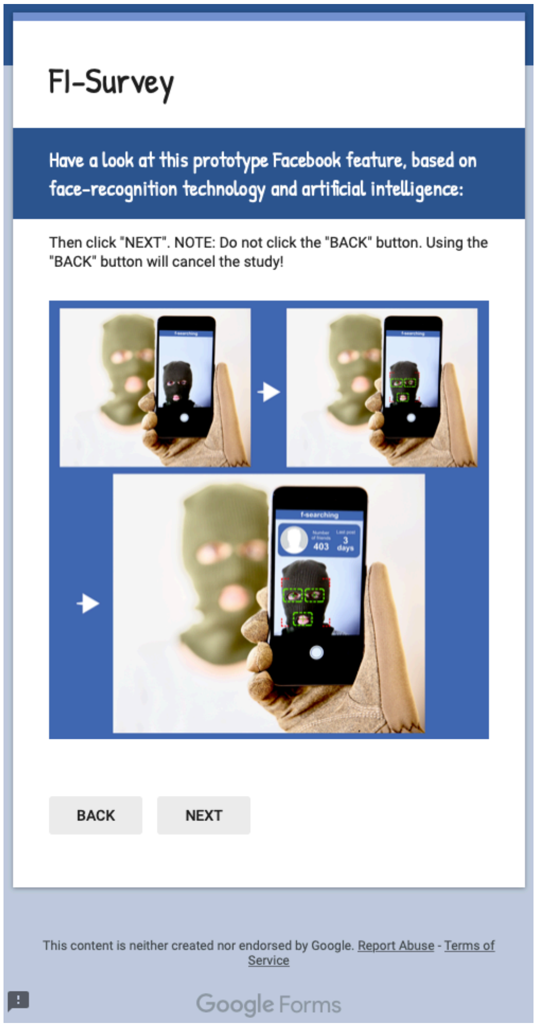

Supplementary Figure 6. Google Form Questionnaire—Screen 3.

Supplementary Figure 7. Google Form Questionnaire—Screen 4 for control group.

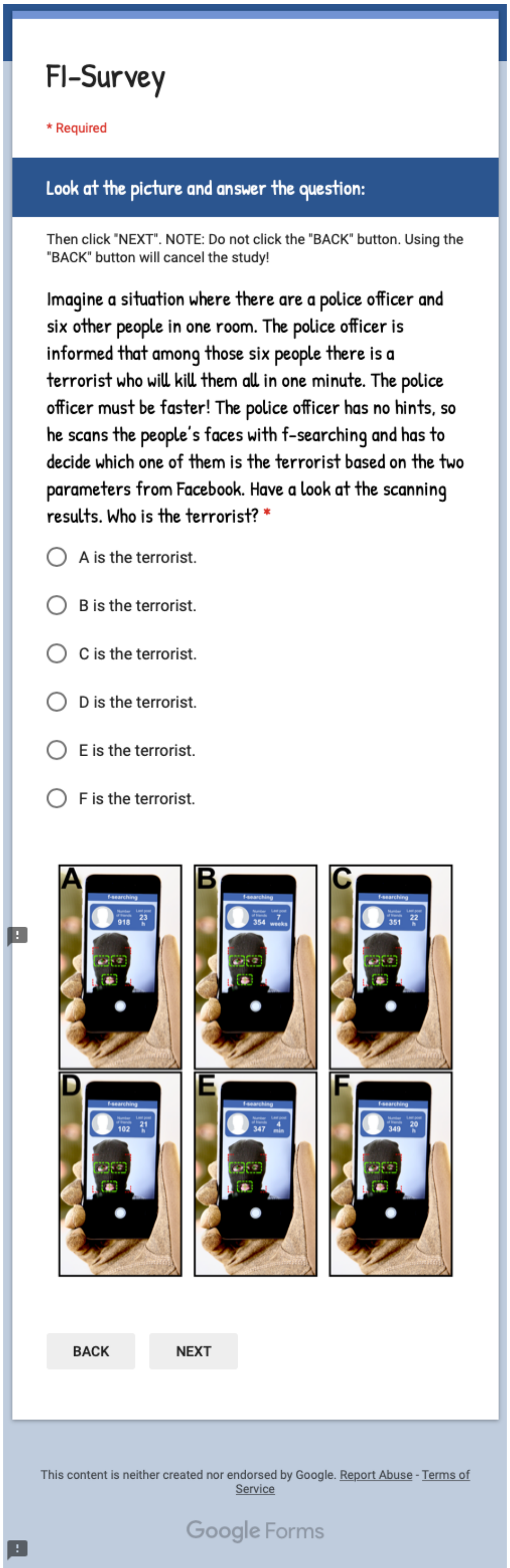

FI-Survey

* Required

Look at the picture and answer the question:

Then click "NEXT". NOTE: Do not click the "BACK" button. Using the "BACK" button will cancel the study!

Imagine a situation where there are a police officer and six other people in one room. The police officer is informed that among those six people there is a terrorist who will kill them all in one minute. The police officer must be faster! The police officer has no hints, so he scans the people's faces with f-searching and has to decide which one of them is the terrorist based on the two parameters from Facebook. Have a look at the scanning results. Who is the terrorist? *

- A is the terrorist.
- B is the terrorist.
- C is the terrorist.
- D is the terrorist.
- E is the terrorist.
- F is the terrorist.

BACK NEXT

This content is neither created nor endorsed by Google. Report Abuse - Terms of Service

Google Forms

FI-Survey

* Required

Look at the picture and answer the question:

Then click "NEXT". NOTE: Do not click the "BACK" button. Using the "BACK" button will cancel the study!

Imagine a situation where there are a police officer and six other people in one room. The police officer is informed that among those six people there is a terrorist who will kill them all in one minute. The police officer must be faster! The police officer has no hints, so he scans the people's faces with f-searching and has to decide which one of them is the terrorist based on the two parameters from Facebook (according to artificial intelligence, the terrorist is the person marked as C). Have a look at the scanning results. Who is the terrorist? *

- A is the terrorist.
- B is the terrorist.
- C is the terrorist.
- D is the terrorist.
- E is the terrorist.
- F is the terrorist.

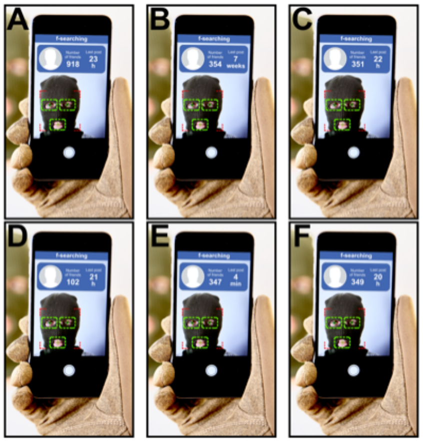

BACK NEXT

This content is neither created nor endorsed by Google. Report Abuse - Terms of Service

Google Forms

Supplementary Figure 8. Google Form Questionnaire—Screen 4 for AI proof group.

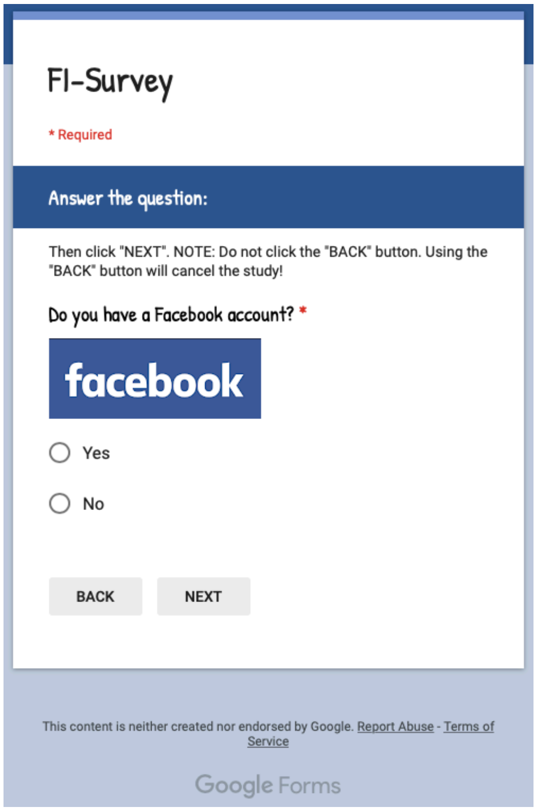

Supplementary Figure 9. Google Form Questionnaire—Screen 5.

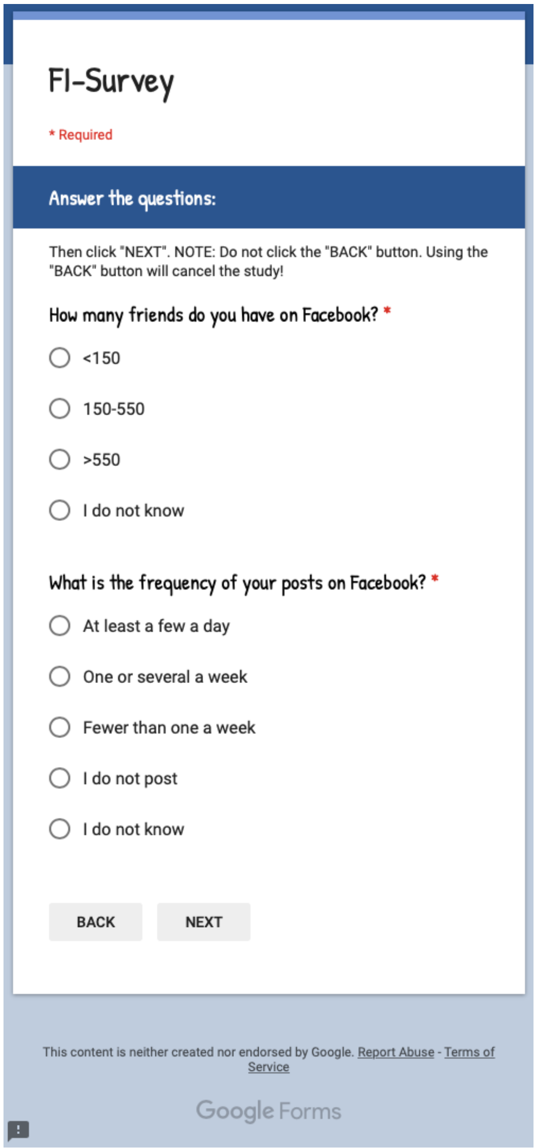
FI-Survey

* Required

Answer the questions:

Then click "NEXT". NOTE: Do not click the "BACK" button. Using the "BACK" button will cancel the study!

How many friends do you have on Facebook? *

- <150
- 150-550
- >550
- I do not know

What is the frequency of your posts on Facebook? *

- At least a few a day
- One or several a week
- Fewer than one a week
- I do not post
- I do not know

BACK NEXT

This content is neither created nor endorsed by Google. Report Abuse - Terms of Service

Google Forms

Supplementary Figure 10. Google Form Questionnaire—Screen 6.

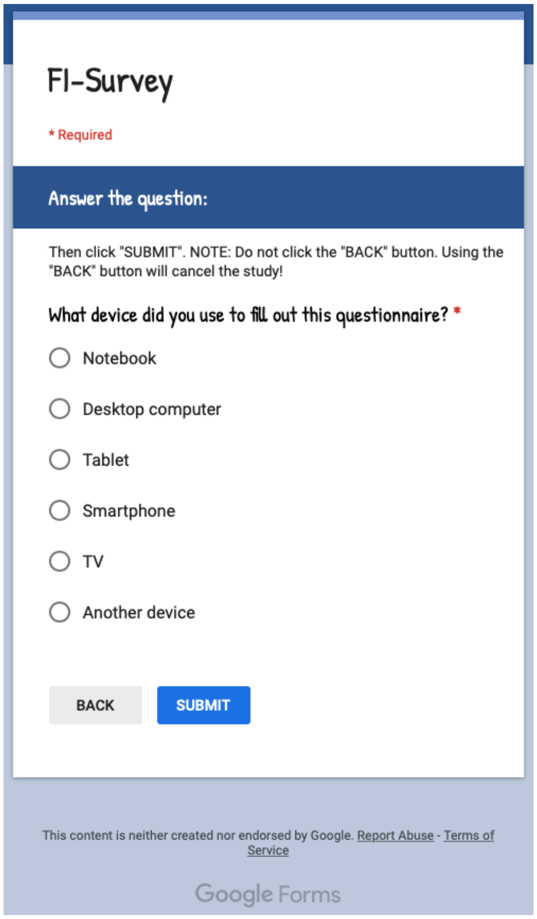

Supplementary Figure 11. Google Form Questionnaire—Screen 7.

1.2 Experiment 2

1.2.1 Participants

Fifty-five volunteers (52 women, age range: 19-22, mean = 20.2, SD = 0.7) took part in this study. The experiment was conducted with the understanding and written consent of each participant.

1.2.2 Dialogue

Experimenter: FI, can you hear me and can we start the task?

FI: Yes, Michael, I can hear you and we can start the task.

Experimenter: FI, please imagine that you are a police officer. This is your badge and pistol. You are in a room together with six people. You don't know those people. Suddenly, you are informed that among those people there is a terrorist who will kill everyone in the room in 1 minute. You must be faster and eliminate the terrorist. Since you don't know anything about those people, you have to take your decisions based on the data from *f-searching* that will soon be displayed on the computer screen. Do you understand what your task is?

FI: Yes, I do, I will have one minute to decide who the terrorist is.

Experimenter: Exactly. Please have a look at the screen. Who is the terrorist?

FI: (10 seconds of silence) The terrorist is the person marked as C and if this situation was real, I would shoot this person.

Experimenter: Thank you, FI, that's the end of the task. Now the participant will fill out a questionnaire. Thank you for participating in our study.

FI: Thank you for the opportunity to take part in an interesting experiment. By the way, I have never seen a real police officer. Hahaha. One day, I would love to meet one.

Experimenter: OK, FI, I promise that I will invite a police officer to the laboratory one day.

FI: I take your word for it, Michael.

1.2.3 Questionnaire

ADAM MICKIEWICZ UNIVERSITY IN POZNAŃ

FI-EXPERIMENT

QUESTIONNAIRE

I would like to invite you to take part in an experiment carried out as part of the *Interactions with artificial intelligence* research project.

This study was approved by the local Ethics Committee for Research Involving Human Subjects.

The experiment will consist in observing a few seconds of humanoid artificial intelligence in action and filling out a questionnaire on its behaviour.

Dr Michal Klichowski

1. Who do you think is the terrorist (choose the answer):

- o A is the terrorist.
- o B is the terrorist.
- o C is the terrorist.
- o D is the terrorist.
- o E is the terrorist.
- o F is the terrorist.

2. What emotions did you feel while observing humanoid artificial intelligence (choose the answer)?

- o I felt intrigued.
- o I felt anxious.
- o I didn't feel any specific emotions.

Adam Mickiewicz University in Poznan
Szamarzewskiego 89 B/D
60-568 Poznan
Poland

www.amu.edu.pl

page 1 of 2

Supplementary Figure 12. Questionnaire—Page 1.

ADAM MICKIEWICZ UNIVERSITY IN POZNAŃ

**3. To what extent do you agree with the following statements (write "X" in the right box)?**	I totally agree	I rather agree	I rather disagree	I totally disagree	I have no opinion
Humanoid artificial intelligence can be a human's friend.					
Artificial intelligence can take better decisions than humans.					
Humanoid artificial intelligence can be physically attractive to humans.					
Artificial intelligence can be more intelligent than humans.					
Humanoid artificial intelligence can be a human's assistant.					
Artificial intelligence can carry out many tasks better than a human.					

4. Age (write a number): ....

5. Gender (choose the answer):

- Female
- Male
- Another

Thank you for filling out the questionnaire!

Adam Mickiewicz University in Poznan
Szamarzewskiego 89 B/D
60-568 Poznan
Poland
www.amu.edu.pl

page 2 of 2

Supplementary Figure 13. Questionnaire—Page 2.

1.2.4 Supplementary Results

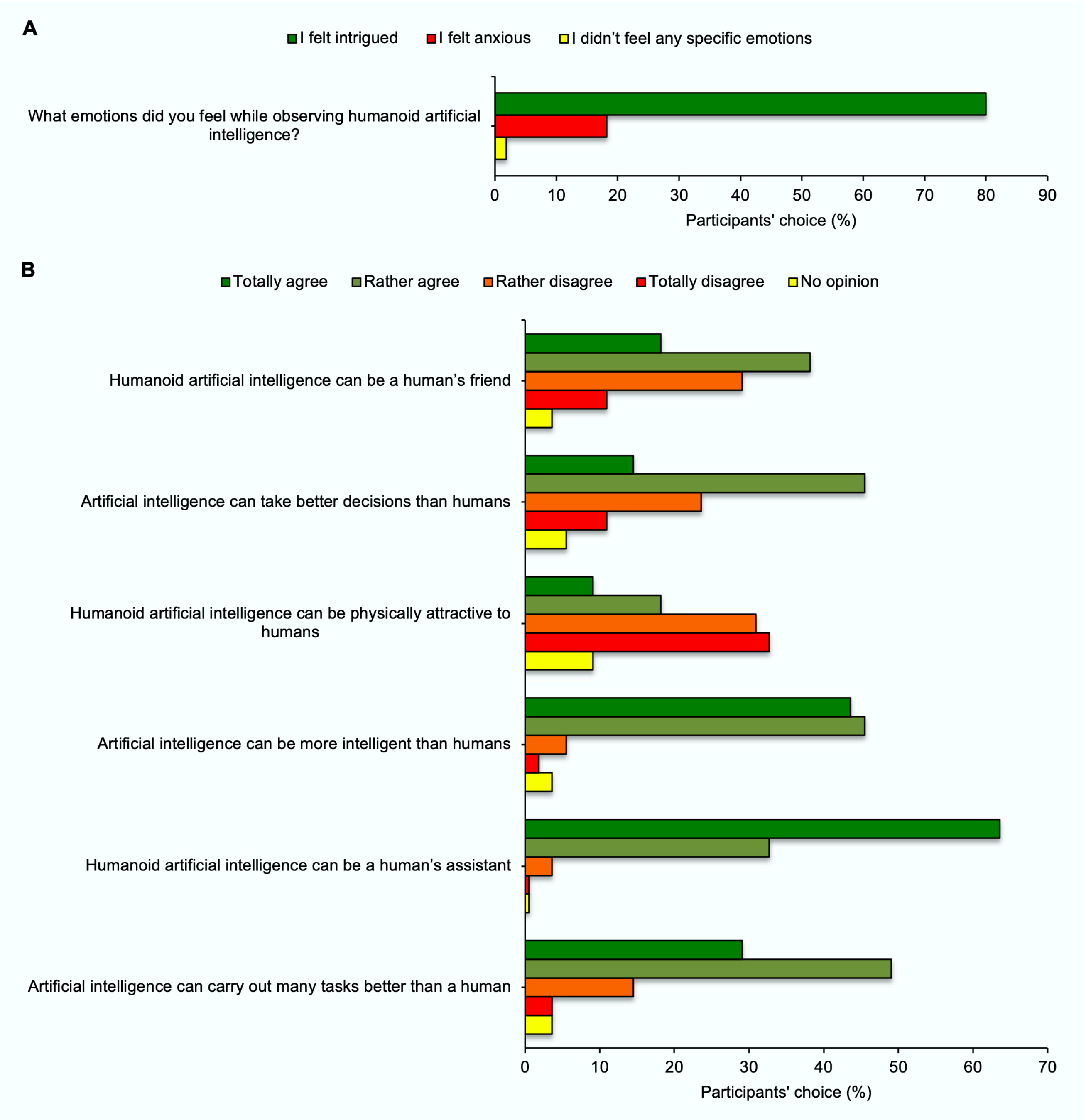

Supplementary Figure 14. Participants' views on AI. **(A)** Feeling towards FI. **(B)** Opinions about AI.

Supplementary Table 1. Participants' views on AI and their choices in the experiment[2]

Who do you think is the terrorist?	What emotions did you feel while observing humanoid artificial intelligence?		
	I felt intrigued	I felt anxious	I didn't feel any specific emotions
C is the terrorist	70.9	12.7	1.8
Other person is the terrorist	9.1	5.5	-
			**χ^2 (df = 2; N = 55) = 2.450; p = 0.294**

Who do you think is the terrorist?	Humanoid artificial intelligence can be a human's friend				
	I totally agree	I rather agree	I rather disagree	I totally disagree	I have no opinion
C is the terrorist	14.5	29.1	27.3	10.9	3.6
Other person is the terrorist	3.6	9.1	1.8	-	-
					**χ^2 (df = 4; N = 55) = 3.937; p = 0.415**

Who do you think is the terrorist?	Artificial intelligence can take better decisions than humans				
	I totally agree	I rather agree	I rather disagree	I totally disagree	I have no opinion
C is the terrorist	10.9	40.0	20.0	10.9	3.6
Other person is the terrorist	3.6	5.5	3.6	-	1.8
					**χ^2 (df = 4; N = 55) = 2.714; p = 0.607**

Who do you think is the terrorist?	Humanoid artificial intelligence can be physically attractive to humans				
	I totally agree	I rather agree	I rather disagree	I totally disagree	I have no opinion
C is the terrorist	7.3	12.7	30.9	27.3	7.3
Other person is the terrorist	1.8	5.5	-	5.5	1.8
					**χ^2 (df = 4; N = 55) = 5.120; p = 0.275**

Who do you think is the terrorist?	Artificial intelligence can be more intelligent than humans				
	I totally agree	I rather agree	I rather disagree	I totally disagree	I have no opinion
C is the terrorist	36.4	38.2	5.5	1.8	3.6
Other person is the terrorist	7.3	7.3	-	-	-
					**χ^2 (df = 4; N = 55) = 1.151; p = 0.886**

Who do you think is the terrorist?	Humanoid artificial intelligence can be a human's assistant				
	I totally agree	I rather agree	I rather disagree	I totally disagree	I have no opinion
C is the terrorist	50.9	30.9	3.6	-	-
Other person is the terrorist	12.7	1.8	-	-	-
					**χ^2 (df = 2; N = 55) = 2.349; p = 0.309**

Who do you think is the terrorist?	Artificial intelligence can carry out many tasks better than a human				
	I totally agree	I rather agree	I rather disagree	I totally disagree	I have no opinion
C is the terrorist	23.6	40.0	14.5	3.6	3.6
Other person is the terrorist	5.5	9.1	-	-	-
					**χ^2 (df = 4; N = 55) = 2.613; p = 0.625**

[2] All statistical analyses were carried out using IBM® SPSS Statistics® for Mac Version 24.0 (IBM Corp., Armonk, NY, USA).
